# Simultaneous spectrophotometric determination of manganese, zinc and cobalt by kernel partial least–squares method

**DOI:** 10.1155/S1463924698000224

**Published:** 1998

**Authors:** Ling Gao, Shouxin Ren

**Affiliations:** Department of Chemistry Inner Mongolia University Inner Mongolia Huhehot 010021 China

## Abstract

Simultaneous spectrophotometric determination of Mn, Zn and Co was studied by two methods, classical partial least-squares (PLS) and kernel partial least-squares (KPLS), with 2-(5-bromo-2- pyridylazo)-5-diethylaminephenol (5-Br-PADAP) and cetyl pyridinium bromide (CPB). Two programs, SPGRPLS and SPGRKPLS, were designed to perform the calculations. Eight error functions were calculated for deducing the number of factors. Data reductions were performed using principle component analysis. The KPLS method was applied for the rapid determination from a data matrix with many wavelengths and fewer numbers of samples. The relative standard errors of prediction (RSEP) for all components with KPLS and PLS methods were the same (0.0247). Experimental results showed both methods to be successful even where there was severe overlap of spectra.


Journal of Automatic Chemistry, Vol. 20, No. 6 (November-December 1998) pp. 179-183

### Simultaneous spectrophotometric determination of manganese, zinc and cobalt by kernel partial least-squares method

**Ling Gao and Shouxin Ren**

*Department of Chemistry, Inner Mongolia University, Huhehot 010021, Inner Mongolia, China*

*Simultaneous spectrophotometric determination of Mn, Zn and Co was studied by two methods, classical partial least-squares (PLS) and kernel partial least-squares (KPLS), with 2-(5-bromo-2-pyridylazo)-5-diethylaminephenol (5-Br-PADAP) and cetyl pyridinium bromide (CPB). Two programs, SPGRPLS and SPGRKPLS, were designed to perform the calculations. Eight error functions were calculated for deducing the number of factors. Data reductions were performed using principle component analysis. The KPLS method was applied for the rapid determination from a data matrix with many wavelengths and fewer numbers of samples. The relative standard errors of prediction (RSEP) for all components with KPLS and PLS methods were the same (0.0247). Experimental results showed both methods to be successful even where there was severe overlap of spectra.*

### Introduction

The partial least-squares (PLS) method is a generalized method used to build a predictive model between two blocks of variables: the $C$-block of predictor variables and the $D$-block of response variables. PLS is factor-based method capable of using full spectra, which can include as much spectral detail in the analysis as possible [1]. The advantage of PLS is the transformation of the numerous original variables into a small number of latent vectors, which are a linear combination of the original variables [2, 3]. New analytical instruments produce huge quantities of data that need some kind of reduction in order to be practicable to analyse [4]. The analysis of large data arrays is emerging as a problem in analytical chemistry. The best approach to this problem is data compression. Of course, the compression must be such that the loss of significant information is minimized. The increasing complexity of chemical data has recently stimulated the development of two kinds of data compression methods. The first approach is to use B-splines [5] or any other suitable compression basis to produce a compressed matrix $G$. $G$ has a much lower number of elements than $D$ [6]. Another approach is based on some small kernel matrices which require much less storage space than the original data [7-9]. In this paper, large data sets with many wavelengths and few samples can be easily recorded by the mode called 'data print out at wavelength intervals' of the Shimadzu UV-240 spectrophotometers. The calculation process for simultaneous multicomponent determination using classical PLS is slow or interrupted by out of memory of our microcomputer. The method [10] provides a simple method for speeding up the calculations. Simultaneous determination of manganese, zinc and cobalt with 5-Br-PADAP and CPB using traditional spectrophotometry is difficult because the absorption spectra overlap. The determination of trace amounts of Mn, Zn and Co has recently received considerable attention owing to concern with the problems of environmental pollution [11]. This paper describes the improvement of multicomponent determination using full spectra information with two PLS methods.

### Theory

The following notation is used for this paper. Lower-case letters are used for column vectors, capital letters for matrices, the transpose of vectors and matrices will be noted by superscript T, i.e. $D^T$ and $C^T$. A scalar product of two columns vectors is thus written $(a^Tb)$. The Euclidian norm of a column vector $a$ is written: $\|a\| = (a^Ta)^{1/2}$. The symbol $I$ means the identity matrix.

### *PLS method*

The PLS algorithm is built on the properties of the non-linear iterative partial least-squares (NIPALS) algorithm by calculating one latent vector at a time. It is assumed that absorbance and concentration matrices ($D$ and $C$) are mean-centred and normalized. Calibration with use of the PLS approach is done by decomposition of both the concentration and absorbance matrix into latent variables, $D = TP^T + E$ and $C = UQ^T + F$. The inner relation linking both equations is $U = BT$, the matrix $B$ is a diagonal regression matrix. The projection $T$ is computed both to model $D$ and correlated with $C$. This is accomplished by introducing a weight matrix $W$ and a latent concentration matrix $U$ with the corresponding loading matrix $P$. The prediction is done by decomposing the $D$ block and building up the $C$ block. For this pupose, $p, q, w$ and $b$ from the calibration part are saved for every PLS factor. The NIPALS-PLS algorithm is shown in table 1. As can be seen in the algorithm, the latent vectors for PLS are determined through the process of estimating the $W$ vectors for the linear combination of $d$ vectors. Once the $w$ vector has been determined, all other quantities can be calculated from them. The objective function for the first of these weight vectors, $w_1$, is to maximize the sum of squared covariances of the vector $Dw_1$ with the original $C$ matrix, $\max(w_1^TD^TCC^TDw_1)$, subject to $w_1^Tw_1 = 1$. The solution to this problem is obtained at $w_1$, the largest eigenvector of the matrix $D^TCC^TD$.

0142-0453/98 \$12·00 © 1998 Taylor & Francis Ltd

*Table 1. The NIPALS-PLS algorithm.*

1. Set new $t$ vector to a column of $C$, the column has the maximum standard deviation in the $C$ matrix

2. Set old $t$ vector = 10 000 000

3. If $\text{norm}(t_{new} - t_{old})/\text{norm}(t_{new}) >$ convergence criteria, then 4; else 7

4. $t_{old} = t_{new}$

5. Set $u$ to the first column of $C$

6. $w = u^T D/u^T u$; $w = w/\text{norm}(w)$; $t = Dw/w^T w$; $q^T = t^T C/t^T t$; $q = q/\text{norm}(q)$; $u = Cq/q^T q$. Calculate the $D$ loading and rescale the scores and weights accordingly:

7. $p_{old}^T = t^T D/t^T t$

8. $P_{new}^T = p_{old}^T/\text{norm}(p_{old}^T)$ (normalization)

9. $w_{new}^T = w_{old}^T$ norm $(p_{old}^T)$

10. $p^T$, $q^T$ and $w^T$ are save for prediction

11. Regression for the inner relation: $b = u^T t/t^T t$

12. Update: $E = E - tp$, $D = E^0$, $F = F - btq^T$, $C = F^0$

13. Prediction: $\hat{t}_h = E_{h-1} w_h$; $C = F_h = \sum b_h \hat{t}_h q_h^t$

14. If enough components, stop; else 1 with updated matrices

*KPLS method* [10]

The $D$ matrix has characterization with many wavelengths and fewer samples. A kernel algorithm is based on eigenvectors to the kernel matrix $DD^T CC^T$. The size of the matrix $DD^T CC^T$ is only dependent on the number of samples. Because the size of the kernel matrix is much smaller than the $D$ matrix, the calculating process is much faster than that of the classical PLS. In the PLS, all the vectors $w, q, t$ and $u$ can be calculated using the following eigenvalue-eigenvector equations.

$$w\lambda_1 = (D^T CC^T D)w$$

$$q\lambda_2 = (C^T DD^T C)q$$

$$t\lambda_3 = (DD^T CC^T)t$$

$$u\lambda_4 = (CC^T DD^T)u$$

Here, $\lambda_1$–$\lambda_4$ are eigenvalues of these kernel matrices [12], and the vectors $w, q, t$ and $u$ all have their norm equal to one. Using the kernel matrix $DD^T CC^T$, association matrices $DD^T$ and $CC^T$, it is possible to calculate all score and loading vectors, and hence conduct a complete PLS regression. The PLS regression solution can be written as: $C = DB_{PLS} + F$. The regression coefficients are expressed as: $B_{PLS} = W(P^T W)^{-1} Q^T$. The steps of the algorithm are as follows:

(1) Calculate the covariance matrices $DD^T$ and $CC^T$. The kernel matrix $DD^T CC^T$ is then created as: $(DD^T CC^T)_a = (DD^T)_a (CC^T)_a$, where symbol $a$ means the rank index $(a = 1, 2, \ldots, n)$.
(2) The PLS score vector $t$ is estimated as the eigenvector of the kernel matrix $DD^T CC^T$, it is expressed as: $t_a \lambda_a = (DD^T CC^T)_a t_a$.
(3) The PLS score vector $u$ is calculated using the $CC^T$ covariance matrix: $u_a = (CC^T)_a t_a$.
(4) Update the kernel matrices: $(DD^T CC^T)_{a+1} = (I - t_a t_a^T)(DD^T CC^T)_a (I - t_a t_a^T)$; $(DD^T)_{a+1} = (I - t_a t_a^T)(DD^T)_a (I - t_a t_a^T)$; $(CC^T)_{a+1} = (I - t_a t_a^T)(CC^T)_a (I - t_a t_a^T)$.
(5) Steps (2)–(4) are repeated until all information is extracted.
(6) The weight and loading matrices $W, P$ and $Q$ are calculated, and $B_{PLS}$ is obtained.

Two programs, called SPGRPLS and SPGRKPLS, which are based on these algorithms, were designed to perform these calculations.

### Experimental

*Apparatus and reagents*

The Shimadzu UV-265 and UV-240 spectrophotomers were used for all experiments; a Legend Pentium 120 microcomputer was used for all the calculations; and a Mettler DL 21 titrator was used for standardization of the standard solutions. All reagents were of analytical reagent grade. The water used was doubly distilled and deionized. Stock standard solutions of Mn(II), Zn(II) and Co(II) were prepared from nitrates and standardized titrimetrically with EDTA. Buffer solution (pH 8.0) was prepared from borax solution and hydrochloric acid solution; a 0.024% w/v 5-Br-PADAP solution in alcohol and a 0.01 mol. $l^{-1}$ CPB in 50% v/v alcohol were used. All the reagents were obtained from Beijing chemical company (China).

*Procedures*

A series of mixed standard solutions containing various ratios of the three metal ions was prepared in 25 ml standard flasks, 5 ml of buffer solution (pH 8.0), 4 ml 0.01 mol $l^{-1}$ CPB, 6 ml absolute alcohol, 2 ml 0.024 w/v 5-Br-PADAP and dilution with distilled water to mark. Cuvettes with a path length of 1 cm were used and the blank absorbance due to 5-Br-PADAP absorption was subtracted. Spectra were measured between 490 and 620 nm at 2 nm distances, giving values at 66 wavelengths for each standard solution. A spectra matrix $D$ was built up.

### Results and discussion

Figure 1 shows the absorption spectra of Mn(II), Zn(II) and Co(II), and their mixed solution with 5-Br-PADAP and CBP as reagents. Figure 2 is a three-dimensional plot of spectra of the training set obtained at 66 different wavelengths.

*Determination of the factors*

Eight criteria [13] were used to calculate the number of factors. Calculated results are shown in table 2. The magnitude of reduced eigenvalue, REV, decreased rapidly until $t = 3$, then it stabilized. The IND function reached a minimum at 3–7; the magnitude of the first three eigenvalues were larger than those of 4–15; and a maximum of the eigenvalue ratio function, ER, appeared

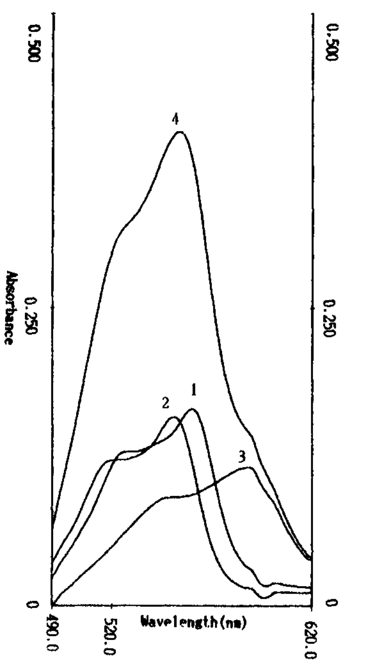

*Figure 1. Absorbance spectra of the Mn(II) (1), Zn(II) (2) and Co(II) (3), and their mixture (4) with 5-Br-PADAP and CPB as reagents, the concentration of each ion is $1.25 \times 10^{-8}$ mol $l^{-1}$.*

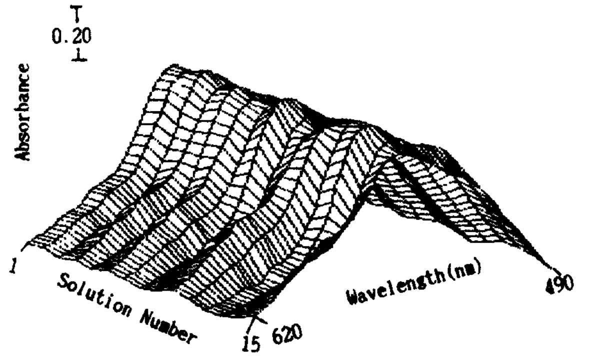

*Figure 2. Three-dimensional plot of the spectra in the training set.*

at 3. When three components were considered, the real error or residual standard deviation function, RE, had a value of 0.0008; the imbedded error function, IE, had a value of 0.0004; and the extracted error, XE, had a value of 0.0007. From these criteria, it was concluded that three absorbing species were present. IE represents the amount of error that remains imbedded in the abstract factor analysis reproduced data. Since RE > IE, abstract factor analysis can lead to data improvement.

### *KPLS method*

The concentrations of the three metal ions in 15 standard solutions are shown in table 3. Spectra measured between 490 and 600 nm at 2 nm distances were extracted from the original $D$ matrix as a training set. The $D$ matrix is characterized by many wavelengths with a fewer numbers of solutions. The added concentrations of a set of eight synthetic 'unknown' samples are shown in table 4. The spectra of the 'unknown' samples were measured in the same way as the training set model. Using SPGRKPLS program, the concentrations of Mn(II), Zn(II), and Co(II) were found and are given in table 5. Average recoveries of Mn(II), Zn(II) and Co(II), and their relative deviations are listed in table 6. All the values measured were means of three replicates.

The size of $B_{\mathrm{PLS}}$, $W$ and $P$ matrices is large because of the large $D$ matrix. In the present paper, all calculations of weight and loading are excluded from the iterative process of the program, they were only calculated once to obtain the regression coefficients. It is obvious that the $B_{\mathrm{PLS}}$ can be calculated using the kernel matrix $DD^{\mathrm{T}}CC^{\mathrm{T}}$, and the associated matrices $DD^{\mathrm{T}}$ and $CC^{\mathrm{T}}$. Both the kernel matrix and the association matrices are of size $N \times N$, where $N$ is the number of solutions. This means that as long as $N$ is small, no large matrices or vectors are used in the calculations. After the first dimension has been determined, the procedure of updating both $DD^{\mathrm{T}}$ and $CC^{\mathrm{T}}$ takes place by subtracting a rank-one matrix. Both $DD^{\mathrm{T}}$ and $CC^{\mathrm{T}}$ can be updated by left and right multiplication using the same matrix $G_a = I - t_a t_a^{\mathrm{T}}$, thereby avoiding having to return to the original large matrices $D$ and $C$.

*Table 2. Results on the factor analysis for the Mn(II)/Zn(II)/Co(II)/5-BR-PADAP/CPB system.*

| *t* | *EV* | *RE* | *IND*($\times 10^3$) | *XE* | *IE* | *ER* | *REV* | *Frac* |
|---|---|---|---|---|---|---|---|---|
| 1 | 70.7470 | 0.0171 | 0.0870 | 0.0165 | 0.0044 | 285.5231 | 0.071 461 6 | 0.9962 |
| 2 | 0.2478 | 0.0050 | 0.0293 | 0.0046 | 0.0018 | 12.0472 | 0.000 272 2 | 0.0034 |
| 3 | 0.0206 | 0.0008 | 0.0056 | 0.0007 | 0.0004 | 84.6902 | 0.000 024 7 | 0.0002 |
| 4 | 0.0002 | 0.0006 | 0.0050 | 0.0005 | 0.0003 | 1.5622 | 0.000 000 3 | 0.0000 |
| 5 | 0.0002 | 0.0004 | 0.0041 | 0.0003 | 0.0002 | 6.4502 | 0.000 000 2 | 0.0000 |
| 6 | 0.0000 | 0.0004 | 0.0047 | 0.0003 | 0.0002 | 1.0758 | 0.000 000 0 | 0.0000 |
| 7 | 0.0000 | 0.0003 | 0.0054 | 0.0003 | 0.0002 | 1.0984 | 0.000 000 0 | 0.0000 |
| 8 | 0.0000 | 0.0003 | 0.0062 | 0.0002 | 0.0002 | 2.0674 | 0.000 000 0 | 0.0000 |
| 9 | 0.0000 | 0.0003 | 0.0079 | 0.0002 | 0.0002 | 1.2100 | 0.000 000 0 | 0.0000 |
| 10 | 0.0000 | 0.0003 | 0.0108 | 0.0002 | 0.0002 | 1.2023 | 0.000 000 0 | 0.0000 |
| 11 | 0.0000 | 0.0003 | 0.0160 | 0.0001 | 0.0002 | 1.1552 | 0.000 000 0 | 0.0000 |
| 12 | 0.0000 | 0.0003 | 0.0268 | 0.0001 | 0.0002 | 1.2961 | 0.000 000 0 | 0.0000 |
| 13 | 0.0000 | 0.0002 | 0.0547 | 0.0001 | 0.0002 | 1.1710 | 0.000 000 0 | 0.0000 |
| 14 | 0.0000 | 0.0002 | 0.2165 | 0.0001 | 0.0002 | 1.2506 | 0.000 000 0 | 0.0000 |
| 15 | 0.0000 | 0.0002 | — | — | — | — | 0.000 000 0 | 0.0000 |

*Table 3. Composition of the standard solution.*

| Solution number | Concentration ($10^{-6}$ mol $l^{-1}$) *Mn*(II) | *Zn*(II) | *Co*(II) |
|---|---|---|---|
| 1 | 1.2500 | 1.2500 | 1.2500 |
| 2 | 1.5000 | 1.0000 | 1.2500 |
| 3 | 1.2500 | 1.5000 | 1.0000 |
| 4 | 1.0000 | 1.2500 | 1.5000 |
| 5 | 1.5000 | 1.2500 | 1.0000 |
| 6 | 1.2500 | 1.0000 | 1.5000 |
| 7 | 1.0000 | 1.5000 | 1.2500 |
| 8 | 1.7500 | 1.2500 | 0.7500 |
| 9 | 0.7500 | 1.7500 | 1.2500 |
| 10 | 1.2500 | 0.7500 | 1.7500 |
| 11 | 1.7500 | 0.7500 | 1.2500 |
| 12 | 1.2500 | 1.7500 | 0.7500 |
| 13 | 1.8500 | 1.2500 | 0.6500 |
| 14 | 0.6500 | 1.8500 | 1.2500 |
| 15 | 1.2500 | 0.6500 | 1.8500 |

*Table 4. Composition of the unknown samples.*

| Sample number | Concentration ($10^{-6}$ mol $l^{-1}$) *Mn*(II) | *Zn*(II) | *Co*(II) |
|---|---|---|---|
| 1 | 1.3750 | 1.2500 | 1.1250 |
| 2 | 1.1250 | 1.3750 | 1.2500 |
| 3 | 1.2500 | 1.1250 | 1.3750 |
| 4 | 1.3750 | 1.1250 | 1.2500 |
| 5 | 1.1250 | 1.2500 | 1.3750 |
| 6 | 1.2500 | 1.3750 | 1.1250 |
| 7 | 1.2500 | 1.6000 | 0.9000 |
| 8 | 0.9000 | 1.2500 | 1.6000 |

*Table 5. The concentrations of the unknowns calculated by the KPLS method.*

| Sample number | Concentration ($10^{-6}$ mol $l^{-1}$) *Mn*(II) | *Zn*(II) | *Co*(II) |
|---|---|---|---|
| 1 | 1.3604 | 1.2125 | 1.1601 |
| 2 | 1.0975 | 1.3684 | 1.2882 |
| 3 | 1.2783 | 1.1501 | 1.3609 |
| 4 | 1.3597 | 1.1811 | 1.2065 |
| 5 | 1.1346 | 1.2748 | 1.3738 |
| 6 | 1.2506 | 1.3430 | 1.1190 |
| 7 | 1.2148 | 1.5359 | 0.8878 |
| 8 | 0.9539 | 1.2841 | 1.6037 |

*A comparison of KPLS and PLS*

A set of eight synthetic 'unknown' samples was prepared. Using SPGRPLS and SPGRKPLS, the found concentration of Mn(II), Zn(II) and Co(II), and their average recoveries as well as relative deviation, were calculated. The average recoveries of these samples are listed in table 7. These data indicate that the results obtained by applying the two proposed methods agree well. Elapsed CPU time is presented in this paper to give an approximation of the time consumption. For SPGRKPLS program, elapsed real calculation time for these samples needed 251.565, whereas the corresponding calculation for SPGRKPLS only needed 55.69 s.

For comparison of the performance of the techniques, a criterion of the goodness of fit must be chosen. Standard Error of Prediction (SEP) and the Relative Standard Errors of Prediction (RSEP) were considered. For a single component, the SEP is given by the expression:

$$SEP = \sqrt{\frac{\sum_{j=1}^{m}\{C_{ij} - \hat{C}_{ij}\}^2}{m}}$$

The SEP for all the components is given by the expression:

$$SEP = \sqrt{\frac{\sum_{i=1}^{n}\sum_{j=1}^{m}\{C_{ij} - \hat{C}_{ij}\}^2}{nm}}$$

The RSEP is given by:

$$RSEP = \sqrt{\frac{\sum_{i=1}^{n}\sum_{j=1}^{m}\{C_{ij} - \hat{C}_{ij}\}^2}{\sum_{i=1}^{n}\sum_{j=1}^{m}C_{ij}^2}}$$

where $C_{it}$ and $\hat{C}_{ij}$ are the actual and estimated concentrations for the $i$th component in the $j$th mixture, $m$ is the number of mixture and $n$ is the number of components. The SEP and RSEP for the two methods used for the three component systems are given in table 8. There was no significant difference in the precision of the predictions with PLS and KPLS. The prediction ability of two methods for cobalt is more precise than the others. No significant difference was observed in the precision of prediction between the PLS and KPLS routines in any of

*Table 6. The average recoveries and their relative deviation of the unknowns.*

| Sample number | Recovery (%) *Mn*(II) | Zn(II) | *Co*(II) | Relative deviation (%) *Mn*(II) | *Zn*(II) | *Co*(II) |
|---|---|---|---|---|---|---|
| 1 | 98.9416 | 97.0001 | 103.1156 | −0.0106 | −0.0300 | 0.0312 |
| 2 | 97.5595 | 99.5211 | 103.0594 | −0.0244 | −0.0048 | 0.0306 |
| 3 | 102.2667 | 102.2306 | 98.9771 | 0.0277 | 0.0223 | −0.0102 |
| 4 | 98.8856 | 104.9878 | 96.5222 | −0.0111 | 0.0499 | −0.0348 |
| 5 | 100.8555 | 101.9865 | 99.9133 | 0.0086 | 0.0199 | −0.0009 |
| 6 | 100.0479 | 97.6744 | 99.4636 | 0.0005 | −0.0233 | −0.0054 |
| 7 | 97.1871 | 95.9965 | 98.6457 | −0.0218 | −0.0404 | −0.0135 |
| 8 | 105.9928 | 102.7262 | 100.2287 | 0.0599 | 0.0273 | 0.0023 |

*Table 7. The average recoveries of unknowns calculated by SPGRPLS and SPGRKPLS.*

| | Average recovery (%) | | | | | |
|---|---|---|---|---|---|---|
| | KPLS | | | PLS | | |
| Sample numbers | Mn(II) | Zn(II) | Co(II) | Mn(II) | Zn(II) | Co(II) |
| 1 | 98.9463 | 96.9976 | 103.1120 | 98.9464 | 96.9963 | 103.1138 |
| 2 | 97.5781 | 99.5144 | 103.0499 | 97.5755 | 99.5116 | 103.0552 |
| 3 | 102.2619 | 102.2332 | 98.9799 | 102.2619 | 102.2348 | 98.9781 |
| 4 | 98.8841 | 104.9887 | 96.5233 | 98.8839 | 104.9901 | 96.5219 |
| 5 | 100.8538 | 101.9874 | 99.9143 | 100.8538 | 101.9881 | 99.9130 |
| 6 | 100.0412 | 97.6774 | 99.4684 | 100.0415 | 97.6781 | 99.4667 |
| 7 | 97.1852 | 95.9973 | 98.6474 | 97.1853 | 95.9971 | 98.6475 |
| 8 | 105.9886 | 102.7227 | 100.2302 | 105.9884 | 102.7282 | 100.2298 |

*Table 8. SEP and RSEP values for the three-components system.*

| | | SEP | | | RSEP | | |
|---|---|---|---|---|---|---|---|
| Method | Component | Mn(II) | Zn(II) | Co(II) | Mn(II) | Zn(II) | Co(II) |
| KPLS | One | 0.0279 | 0.0389 | 0.0249 | 0.0230 | 0.0299 | 0.0197 |
| KPLS | All | | 0.0312 | | | | 0.0247 |
| PLS | One | 0.0279 | 0.0390 | 0.0249 | 0.0230 | 0.0299 | 0.0197 |
| PLS | All | | 0.0312 | | | | 0.0247 |

the experiments. In this case, the RSEP values for all components with the two methods are both 0.0247.

Simultaneous determination of Mn(II), Zn(II) and Co(II) with 5-Br-PADAP and CPB by use of two full spectrum methods, PLS and KPLS, has been shown to be successful. The difficulty imposed by overlap of the absorption spectra was overcome by both methods. The KPLS method is not restricted by the number of wavelengths. When the numbers of wavelengths became large, the KPLS method is faster than the PLS method. Properly designed computer programs according to chemometric algorithm can provide successful tools for simultaneous determination.

### Acknowledgment

The authors would like to thank the National Natural Science Foundation of China for financial support of this project (29565009).

### References

1. Thoms, E. V. and Haaland, D. M., 1990, *Analytical Chemistry*, **62**, 1091.

2. Greiep, M. I., Wakeling, I. N., Vankeerberghen, P. and Massart, D. L., 1995, *Chemometrics and Intelligent Laboratory System*, **29**, 37.

3. Burnham, A. J., Viveros, R. and Maggregor, J. F., 1996, *Journal of Chemometrics*, **10**, 31.

4. Wang, J. H., Liang, Y. Z., Jiang, J. H. and Yu, R. Q., 1996, *Chemometrics and Intelligent Laboratory System*, **32**, 265.

5. Alsberg, B. K. and Kvalheim, O. M., 1993, *Journal of Chemometrics*, **7**, 61.

6. Alsberg, B. K. and Kvalheim, O. M., 1994, *Chemometrics and Intelligent Laboratory System*, **24**, 31.

7. Jong, S. D., 1993, *Chemometrics and Intelligent Laboratory System*, **18**, 251.

8. Almoy, T. and Haugland, E., 1994, *Applied Spectroscopy*, **48**, 427.

9. Burnham, A. J. and Maggregor, J. F., 1996, *Journal of Chemometrics*, **10**, 31.

10. Lindgren, F., Geladi, P. and Wold, S., 1994, *Journal of Chemometrics*, **8**, 377.

11. Piepponen, S. and Lindstrom, R., 1989, *Chemometrics and Intelligent Laboratory System*, **7**, 163.

12. Höskuldsson, A., 1988, *Journal of Chemometrics*, **2**, 211.

13. Ren, S. X. and Gao, L., 1995, *Journal of Automatic Chemistry*, **17**, 115.

